# Deciphering the Clinical Implications of Concurrent Chromosome 7 Gain and Chromosome 10 Loss in Glioblastoma: A Scoping Review

**DOI:** 10.3390/brainsci16010060

**Published:** 2025-12-31

**Authors:** Edgar G. Ordóñez-Rubiano, Alexandra Ramos-Márquez, Raul F. Vega-Alvear, Clara Ruiz-Forero, Antonia Cadavid-Cobo, Santiago Fuentes-Tapias, Pedro Andrade-Andrade, Alba L. Cómbita, César Payán-Gómez, Rafael Parra-Medina, Diego F. Gómez, Juan F. Ramón, Fernando Hakim

**Affiliations:** 1 Department of Neurosurgery, Hospital Universitario Fundación Santa Fe de Bogotá, Bogotá 110111, Colombia; 2 Department of Neurosurgery, Hospital de San José—Fundación Universitaria de Ciencias de la Salud, Bogotá 110221, Colombia; 3 Facultad de Medicina, Universidad Nacional de Colombia, Bogotá 11321, Colombia; 4 School of Medicine, Universidad del Norte, Barranquilla 080001, Colombia; 5 School of Medicine, Universidad de los Andes, Bogotá 111711, Colombia; 6 Grupo de Investigación en Biología del Cáncer, Instituto Nacional de Cancerología (INC), Bogotá 111511, Colombia; 7 Grupo de Investigación Traslacional en Oncología, Instituto Nacional de Cancerología (INC), Bogotá 111511, Colombia; 8 Departamento de Microbiología, Facultad de Medicina, Universidad Nacional de Colombia, Bogotá 111321, Colombia; 9 Dirección Académica, Universidad Nacional de Colombia, Sede de La Paz, La Paz 201501, Colombia; 10 Departamento de Patología, Instituto Nacional de Cancerología (INC), Bogotá 110221, Colombia; 11 Instituto de Investigación, Fundación Universitaria de Ciencias de la Salud (FUCS), Bogotá 110221, Colombia

**Keywords:** glioblastoma, glioma, chromosome 7 gain, chromosome 10 loss, +7/−10 signature

## Abstract

**Background/Objectives**: Combined chromosome 7 gain and chromosome 10 loss (+7/−10) is the most frequent cytogenetic alteration and a defining diagnostic criterion for isocitrate dehydrogenase wild-type (IDHwt) glioblastoma. Despite the association with poor prognosis, its clinical and therapeutic significance remains unclear. We aim to systematically review its clinical significance, focusing on prevalence, prognostic value, and potential association with therapeutic resistance in adult patients. **Methods**: PubMed, Embase, CENTRAL, Scopus, EBSCOhost, and Web of Science were searched from inception to April 2025, using controlled vocabulary and free-text terms. Eligible studies included adult glioblastoma with molecular confirmation of combined chromosome 7 gain and chromosome 10 loss and reported survival or treatment response. Quality was assessed qualitatively, and findings were synthesized descriptively. **Results**: Of 3249 records, 5 observational studies (523 patients) were included. The signature was present in 60% to 70% of glioblastoma cases and frequently co-occurred with epidermal growth factor receptor amplification and telomerase reverse transcriptase promoter mutations. This alteration was consistently associated with shorter survival (mean, 8–70 weeks) compared with tumors lacking the alteration (19–170 weeks). In one study, the signature was more common in radioresistant tumors (9/20 vs. 1/10). Molecular evidence suggests that this alteration arises early in tumorigenesis. **Conclusions**: The +7/−10 cytogenetic alteration, common in glioblastoma, is frequently associated with aggressive clinical behavior. While exploratory data suggest a possible association with radiotherapy response, current evidence is insufficient to establish a predictive or therapeutic role. Its principal clinical value lies in diagnosis, molecular classification, and risk stratification. Incorporating cytogenetic testing for this alteration into routine glioblastoma workup may improve risk stratification and guide individualized management.

## 1. Introduction

Glioblastoma (GBM) is a highly aggressive brain tumor characterized by a genetic and molecular component that determines its classification and disease progression [1]. A common genomic alteration is the co-occurrence of chromosome 7 gain and chromosome 10 loss (+7/−10) [2], incorporated into the 5th version of the World Health Organization (WHO) classification of the tumors of the central nervous system (CNS) to define isocitrate dehydrogenase wildtype (IDHwt) GBM [2,3,4]. These variations reflect high genomic instability [1]. Its presence leads tumors previously classified as high-grade glioma (astrocytomas IDHwt, grades 2 and 3) to acquire a GBM status [3].

Although this genetic signature confers a diagnosis of GBM to astrocytomas IDHwt grades 2 and 3 and is associated with an aggressive clinical course, its clinical implications remain unclear among GBM patients [5]. The heterogeneity of therapeutic response, tumor aggressiveness, and overall survival (OS) has not yet been studied. Despite the well-established association of +7/−10 with GBM, no prior study has systematically compared GBM patients exhibiting this cytogenetic profile and those without it in terms of clinically relevant outcomes. This scoping review aims to address this gap by focusing exclusively on GBM, thereby providing a more nuanced understanding of the prognostic relevance of these chromosomal alterations within this distinct tumor entity.

## 2. Materials and Methods

This scoping review was conducted following the PRISMA-ScR (Preferred Reporting Items for Systematic Reviews and Meta-Analysis Extension for Scoping Reviews) Checklist (Supplemental Material). The protocol for this scoping review was registered in the Open Science Framework database (DOI: 10.17605/OSF.IO/ZBUAY).

### 2.1. Eligibility Criteria

Studies involving adults with GBM with molecular and genetic analysis to identify the +7/−10, reporting at least one clinical outcome, such as OS and/or therapeutic response. The types of studies sought were clinical trials, cohort studies, descriptive studies, case series, and/or case reports. No restrictions were applied for publication date or language if translation was available.

Exclusion criteria were pediatric patients; midline GBM, brainstem GBM, GBM with oligodendroglial component, or other variants; studies involving separate chromosome 7 gain or chromosome 10 loss (+7/−10), or only one chromosome arm abnormality. Non-peer-reviewed publications, journal supplements, editorials, letters, technical notes, conference abstracts, or non-human participant studies were not considered.

### 2.2. Information Sources and Search Strategy

A systematic literature search was conducted across databases and search engines (PubMed, Embase, CENTRAL, Scopus, EBSCOhost, and Web of Science) up to 20 April 2025. Keywords using MeSH and DeCS descriptors, as well as free terms, were applied to search for “whole chromosome 7 gain”, “whole chromosome 10 loss”, “chromosome 7 gain/10 loss”, and “glioblastoma”.

### 2.3. Selection of Sources of Evidence

Zotero and Rayyan (https://www.rayyan.ai/, accessed from 1 January 2023 to 31 October 2025) were used to manage references and remove duplicates, and Rayyan for article selection. Four authors in two pairs screened titles and abstracts, each pair reviewing half of the references. Full-text reviews were then conducted independently by all four authors. Discrepancies were resolved between pairs. Reference lists of included studies were also reviewed to identify additional eligible studies.

### 2.4. Data Charting Process and Data Items

Four authors, working in pairs, independently extracted data from the included studies, dividing the references equally. Extracted information was compiled in a spreadsheet. It included: study details (first author, title, year, journal, country, and funding), population characteristics (sample size, mean age, and sex) tumor characteristics (diagnosis, location, imaging findings, molecular and genetics features, EGFR amplification status, details about the chromosome 7/chromosome 10 abnormalities, and the molecular method for analysis), management (surgery, chemotherapy and/or radiotherapy), and outcomes. Discrepancies were resolved collaboratively through discussion among the authors.

### 2.5. Synthesis of Results

The data extracted from the included studies was synthesized through a qualitative analysis, focusing on key themes across the literature. The results are presented descriptively, utilizing tables to systematically organize and summarize the main findings.

## 3. Results

### 3.1. Selected Studies

**Figure 1:**
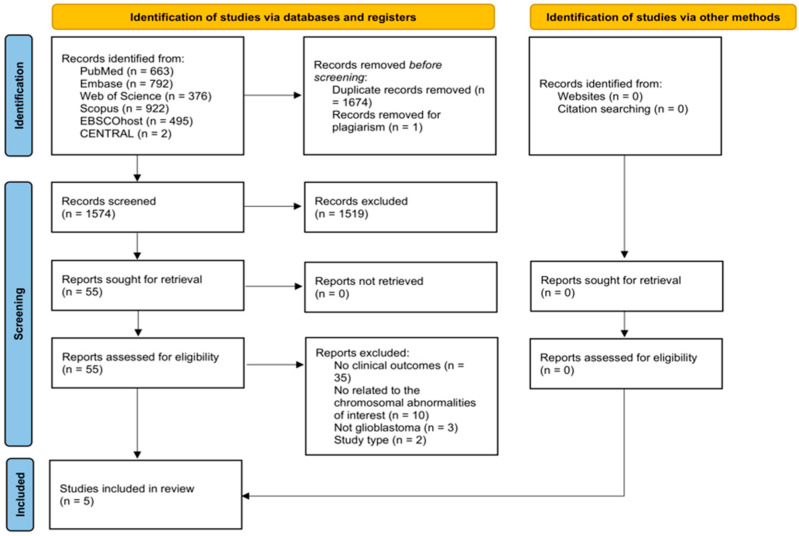
Preferred Reporting Items for Systematic Reviews and Meta-Analysis Extension for Scoping Reviews flow diagram of study selection.

A total of 3249 articles were identified through the systematic search. After removing duplicates, 1574 studies were screened by title and abstract. 54 articles were sought for retrieval and were included for full-text review. Five studies were included in this review [6,7,8,9,10] (Figure 1).

### 3.2. Study Characteristics

All five included observational studies [6,7,8,9,10] were from the United States [6,10], Turkey [7], Germany [8], and Spain [9]. Four studied the correlation of the +7/−10 alteration among GBM patients and its impact on OS [6,7,8,9], and the other one on differential response to radiotherapy according to the presence of the co-occurrence [10]. A summary of the included studies is presented in Table 1 and Table 2.

### 3.3. Study Outcomes and Synthesis of Results

#### 3.3.1. Chromosome 7 Gain and Chromosome 10 Loss, and OS

López-Ginés et al. [9] studied 25 newly diagnosed GBM cases using fluorescence in situ hybridization (FISH) to assess chromosomal status and EGFR amplification. 58% of patients (14/25) had trisomy or polysomy of chromosome 7 and monosomy of chromosome 10. Of these, 28% (7/25) exhibited this chromosomal alteration alongside EGFR amplification. Overall, 52% of patients had EGFR gene amplification alone, and the +7/−10 was the most frequent alteration. Survival analysis showed that patients with +7/−10, regardless of EGFR status, had shorter survival times than those without these alterations (mean survival: 36 ± 3.7 weeks vs. 48 ± 4.7 weeks). These findings suggest a possible association between the +7/−10 alteration and poorer prognosis; however, the limited sample size and lack of statistical significance preclude definitive conclusions. It may represent an early event in tumorigenesis, occurring before EGFR gene amplification, which might be a later and independent event.

Arslantas et al. [7] conducted genomic profiling of primary GBMs and chromosomal abnormalities with potential prognostic significance. The most common alteration in patients with shorter OS was the complete loss of chromosome 10, however tumors showing both +7/−10 alteration, particularly in the 7p11–p13 region, were consistently associated with poor outcomes, hence limiting the specificity of this signature as an independent prognostic marker Five cases exhibited the +7/−10 alteration, with a mean survival of just 5.0 ± 3.1 months, compared to the overall average survival of 10.0 ± 5.6 months across all patients. This suggests that the +7/−10 alteration profile may serve as a marker of poor prognosis among GBM patients

Stichel et al. [8] evaluated the prognostic impact of the +7/−10 alteration across glioma subtypes. Survival analysis on 939 IDHwt gliomas showed that patients with the +7/−10, +7/−10q, or +7q/−10 alterations had significantly worse OS. Although the study did not directly compare GBM patients with and without the +7/−10 signature, a subset analysis of 167 patients, classified as GBM based on methylation profiling, revealed that 52 patients did not carry the +7/−10 alteration, but showed survival curves nearly identical to those of the 115 GBM patients who did. This finding emphasizes that while the +7/−10 signature may be associated with poor prognosis, it is not strictly necessary for observing poor survival rates in GBM.

Finally, Nair et al. [6] in their survival analysis of GBM patients from The Cancer Genome Atlas (TCGA) cohort, stratified by chromosome 7 and 10 copy number status, revealed a marked difference in overall prognosis depending on the presence of aneuploidy. Patients with +7/−10 tumors exhibited the poorest OS, with most dying within 70 weeks. In contrast, patients without either alteration demonstrated relatively prolonged survival, in some cases exceeding 170 weeks. Intermediate outcomes were observed in patients with isolated alterations. However, only four patients lacked chromosomal alterations, substantially limiting the robustness of comparisons and precluding definitive conclusions.

#### 3.3.2. Chromosome 7 Gain and Chromosome 10 Loss, and Radiotherapy Response

The study by Huhn et al. [10] explored the relationship between chromosomal abnormalities and radiotherapy response in GBM using comparative genomic hybridization. A total of 30 frozen tumor samples were analyzed and categorized into radiation-resistant (n = 20) and radiation-sensitive (n = 10) groups based on contrast enhancement changes following treatment. The +7/−10 signature was identified in 9 out of 20 resistant tumors, compared to only 1 out of 10 in the radiosensitive group. Although the +7/−10 alteration was more frequently observed in radiation-resistant tumors, no statistically significant association was demonstrated. These findings should be interpreted as hypothesis-generating only; worth mentioning that this specific chromosomal alteration and resistance to radiotherapy warrant further investigation in larger, controlled cohorts. The authors proposed that a higher frequency of copy number alterations (CNAs), including +7/−10, in the radioresistant group may indicate increased genomic instability, potentially contributing to a phenotype more resilient to radiation.

## 4. Discussion

### 4.1. 7/10 Signature Prevalence and Its Relationship with GBM

This review assessed the clinical significance of the +7/−10 signature in GBM. Across the five included studies, prevalence estimates of the +7/−10 signature ranged from approximately 60% to 70% of GBM cases, although direct comparisons are limited by differences in cohort composition and detection methodology [6,7,8,9,10]. Gains of chromosome 7 alone were highly frequent, but losses of chromosome 10 were almost always found in conjunction with chromosome 7 gains. Other chromosomal alterations included amplification of 12q regions, isolated loss of 1p, 9p, 17p, and 19q, and gains involving chromosomes 19 and 20. However, these were less frequent than the hallmark +7/−10 [7,10]. These findings emphasize the predominance of +7/−10 as a defining feature of GBM genomic architecture, underscoring their central role in tumor initiation and progression.

Genomic instability drives cancer initiation and progression, ranging from small DNA sequence mutations to large-scale chromosomal abnormalities [11,12]. +7/−10 alteration in GBM represents a recurrent form of numerical chromosomal instability, which is a well-established consequence of centrosome amplification and mitotic checkpoint failure [13,14]. Centrosomes organize microtubules for bipolar spindle formation in mitosis [12], but amplification in tumors leads to multipolar spindles, causing chromosome missegregation and aneuploidy [12,13,14]. Cancer cells cluster extra centrosomes into pseudo-bipolar spindles, preserving viability while keeping chromosomal instability [12,15].

In parallel, defects in the spindle assembly checkpoint (SAC), mediated by kinases such as Aurora A and B, allow mitotic errors and promote the evolution of tetraploid intermediates [16,17]. These intermediates, particularly in TP53-deficient contexts, bypass checkpoints and proliferate with increased centrosome and genomic content, propagating karyotypic chaos [12]. This interplay between centrosome amplification and SAC dysfunction drives the selection of specific, clonally advantageous aneuploidies such as the +7/−10, which confer both survival benefits and proliferative potential.

Other mechanisms contribute to the development of aneuploidy in cancer. Mitotic spindle assembly defects, particularly errors in microtubule–kinetochore attachments, can lead to lagging chromosomes and subsequent missegregation during cell division [18,19]. Likewise, kinetochore malfunctions, arising from structural abnormalities or disrupted protein complexes, impair the proper attachment and alignment of chromosomes on the spindle apparatus, further increasing the risk of aneuploid progeny [19,20]. Dysfunction of the cohesin complex, essential for maintaining sister chromatid cohesion until anaphase, can result in premature chromatid separation and chromosomal instability [21,22]. These mechanisms often act in concert with the centrosome and checkpoint abnormalities, collectively driving the chromosomal imbalances characteristic of tumors with high chromosomal instability, such as GBM.

There are a few studies investigating the intricate mechanisms underlying the occurrence of this chromosomal phenomenon. Körber et al. [23] studied the mechanism of driving GBM evolution by reconstructing the phylogenetic trajectories of primary and recurrent tumors. Their study demonstrated that chromosome 7 gain and chromosome 10 loss are among the earliest clonal events, often occurring together at tumor initiation, even years before clinical diagnosis. These large-scale chromosomal alterations establish a common evolutionary path across IDHwt GBM, preceding other driver mutations such as telomerase transcriptase reverse promoter (TERTp) mutations. This suggests that the +7/−10 reflects essential early steps in tumorigenesis rather than consequences of later selective pressures, highlighting their relevance as stable biomarkers and potential early therapeutic targets.

Building on these findings, Nair et al. [6] provided important insights into the early genomic events by exploring the functional relationship between chromosome 10 loss and 7 gain, proposing that their frequent co-occurrence is driven by a synthetic rescue mechanism. Loss of chromosome 10 removes key tumor suppressor genes such as PTEN, ANXA7, and KLF6 [24,25,26,27], which, while favoring tumor progression, also disrupt essential cellular processes and impose a significant fitness cost. To survive this, tumor cells upregulate compensatory genes, often oncogenes, on chromosome 7. These include EGFR [28], which can functionally rescue PTEN loss; and MET [29], which compensates for ANXA7 loss. This phenomenon reflects an adaptive, not random, evolutionary response, whereby chromosome 7 gain mitigates the detrimental effects of chromosome 10 loss through a distributed compensatory network.

**Figure 2:**
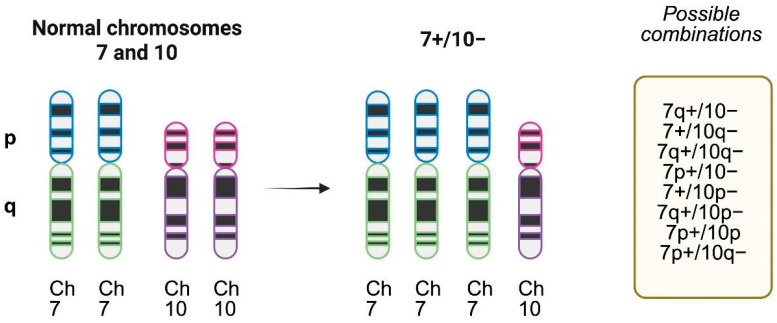
Chromosome 7 gain and chromosome 10 loss patterns of alterations. Complete chromosome 7 gain (trisomy) and 10 loss (monosomy) is the most frequent pattern, but several possible combinations in the short (p) or long arm (q) exist and have been described in molecular studies. Created with www.biorender.com.

**Figure 3:**
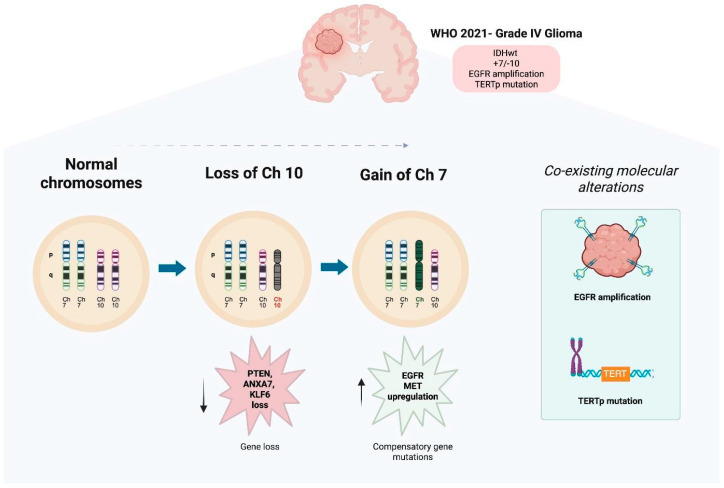
Key Mechanisms in GBM Evolution: +7/−10 Signature, EGFR Amplification, and TERTp Mutation. Created with www.biorender.com.

Such interplay illustrates how chromosomal aneuploidy in GBM is not merely a byproduct of genomic instability but a dynamic and selective mechanism enhancing tumor fitness. Together, these studies emphasize that chromosome 7 gain and 10 loss are both foundational events and functional adaptations in GBM pathogenesis, with important implications for understanding tumor evolution and guiding therapeutic development. Several possible chromosomal alterations for chromosomes 7 and 10 exist, as seen in Figure 2. This interplay between the +7/−10 along with co-occurring EGFR amplification and TERTp mutations is illustrated in Figure 3, highlighting their role as early and functionally compensatory events in GBM pathogenesis.

Progression from normal chromosomal configuration to the characteristic gain of chromosome 7 and loss of chromosome 10 (+7/−10) is observed in GBM. The loss of chromosome 10 leads to the deletion of key tumor suppressor genes, including PTEN, ANXA7, and KLF6. There is impaired tumor suppressive functions, and as a compensatory response, there is a gain of chromosome 7, which results in overexpression of oncogenes like EGFR and MET, which promote cell proliferation and survival. These early and co-occurring chromosomal events contribute to genomic instability and establish a clonally advantageous profile that underlies GBM initiation and progression. EGFR amplification and TERT promoter (TERTp) mutations frequently co-occur with the +7/−10 signature, defining a distinct subgroup of high-risk gliomas. This cytogenetic pattern is integrated into the 2021 WHO classification of CNS tumors [30] for the diagnosis of grade IV IDH-wildtype glioblastoma, even in tumors lacking overt high-grade histological features.

Amalfitano et al. [31] analyzed chromosomes 7 and 10 via FISH in 64 GBM (44 primary, 20 secondary), finding similar rates of chromosome 7 gain and 10 loss across both subtypes. Combined +7/−10 alterations appeared in 68% of primary and 70% of secondary tumors. Notably, 14.3% of primary GBM with chromosome 10 loss retained chromosome 7 disomy, unlike secondary cases. This led the authors to suggest that chromosome 10 loss may precede chromosome 7 gain, especially in secondary GBM.

### 4.2. Clinical Implications of the +7/−10 Signature

The +7/−10 alteration is one of the most frequent and significant alterations in GBM, especially among IDHwt tumors [6,32]. Its presence is associated with poor OS [32]. Currently, it is part of the defining molecular features of GBM, even in tumors with lower-grade histology [32]. Multiple studies have shown [33,34,35] that patients with IDHwt astrocytomas who have this cytogenetic pattern experience clinical outcomes indistinguishable from those of conventional GBM, supporting their reclassification as GBM based on molecular findings, even if they were classified as low-grade histologically [32]. Partial chromosomal alterations, such as 7q gain combined with 10q loss, appear to reflect the clinical relevance of changes in the entire arm [32]. Additionally, it has also been detected in radiation-resistant tumors, suggesting a possible association with treatment failure, primarily in response to radiotherapy [10]. Therefore, it is of utmost importance to incorporate cytogenetic testing into standard diagnostic testing, as early identification of this pattern has direct implications for both prognosis and treatment planning.

On the other hand, other key molecular alterations that coexist with chromosome 7 gain and chromosome 10 loss are EGFR amplification and TERTp mutation. These two co-occurrences have shown specificity of up to 99.4% for IDHwt GBM and are absent in non-GBM entities [3,6]. EGFR amplification, typically mapped to 7p11.2, tends to appear after complete chromosome 7 gain and is associated with increased pathway activation and tumor proliferation [6,8]. Although EGFR amplification is the most specific of these markers, its sensitivity is limited, so it should be interpreted in conjunction with the +7/−10 and TERTp mutation status [3]. TERTp mutations, on the other hand, are more prevalent but less specific [8]. Taken together, the +7/−10, EGFR amplification, and TERTp mutation define a subgroup of high-risk gliomas that warrants precise classification and could benefit from individualized therapeutic strategies [6,8].

Furthermore, other alterations, along with the +7/−10, can shape clinical behavior and treatment response, such as codeletions involving 9p23–24 and 13q14 (regions that include CDKN2A/B and RB1), that are frequently found in older patients and have been linked to radioresistance [10]. These deletions possibly contribute to impaired cell cycle control and reduced apoptotic potential. Other recurrent alterations include gains in chromosomal regions such as 7q, 19p, 1p, 4q, and 12q, which are thought to promote genomic instability and more aggressive phenotypes [6]. Some of these combinations may reflect rescue interactions, where gains of oncogenes on chromosome 7 (e.g., EGFR, MET, BRAF) can compensate for losses on chromosome 10 (e.g., PTEN, ADARB2), collectively improving tumor survival [6].

### 4.3. Future Perspectives

The +7/−10 signature has shown utility as a molecular hallmark for the diagnosis of IDHwt GBM, and upgrading the diagnosis of IDHwt astrocytomas (grades 2 and 3) to GBM, as seen in the 2021 WHO CNS tumor classification [30], in spite of nuclear or ambiguous histopathological features [3,8,36]. Looking toward the future, probable implications regarding treatment outcomes and patient stratification can be expected, along with a more prominent role of molecular studies for earlier aggressive tumor recognition. Current studies are being carried out to differentiate between the partial or whole chromosome alterations in diagnosis and prognosis [7,28], as well as to elucidate the interaction between these molecular changes with other genetic alterations or biomarkers, to generate improved grading and risk groups [7,37]. Furthermore, targeted therapy and non-invasive monitoring of these genetic alterations could also be implemented in the future [3].

### 4.4. Limitations

Despite the limitations of this scoping review, it provides meaningful insights into the prognostic significance of the +7/−10 in GBM. The small number of studies included, reduced study samples, and the variability in study designs warrant careful interpretation of the findings. Evidence suggests the co-occurrence is associated with poorer survival and may be a biomarker for aggressive GBM.

Future research should aim to address these limitations by expanding the sample size to enhance the statistical power for the generalizability of the results. Additionally, standardization of methodologies, particularly in chromosomal analysis, is essential for consistency across studies. Longitudinal, prospective cohort studies and randomized clinical trials are needed to further explore the prognostic implications of the +7/−10, as well as its potential role in resistance to therapies such as radiotherapy and/or chemotherapy. Ultimately, these efforts will help refine our understanding of GBM progression and improve patient outcomes through more personalized treatment approaches.

## 5. Conclusions

The +7/−10 signature is a frequent cytogenetic alteration in GBM and is consistently associated with poor survival across multiple cohorts. While biological and exploratory clinical data suggest a possible link with treatment resistance, current evidence remains insufficient to establish a predictive role. Its principal clinical value at present lies in diagnosis, molecular classification, and risk stratification. While not exclusive to GBM, the +7/−10 signature remains a valuable prognostic marker for high-grade glioma. Future research should explore its role in therapeutic resistance and its potential as a target for personalized treatment strategies.

## Figures and Tables

**Table 1:** Overview of included studies.

Authors	Country and Year	Study Design	Sample Size	GBM Subtype	Method	Key Findings
Arslantas et al. [7]	Turkey, 2004	Observational study	20	Primary GBM	CGH	Loss of chromosome 10 and chromosome 7/7p amplification may serve as specific molecular markers associated with poor prognosis among patients with primary GBM.
Lopez-Gines C et al. [9]	Spain, 2005	Observational study	25	Newly diagnosed GBM	FISH	Survival rate analysis demonstrated lower survival rates in patients with monosomy 10, trisomy 7, and monosomy associated with trisomy 7. EGFR gene amplification appears to be independent of this co-occurrence.
Stichel et al. [8]	Germany, 2018	Observational study	167	Primary GB	DNA methylation	Chromosome +7/−10 signature associated with worse survival in IDH-wildtype gliomas. However, GB-like survival is also observed in patients without the signature, suggesting it is not essential for poor prognosis in molecularly defined GBM.
Nair NU et al. [6]	USA, 2024	Observational study	141	Primary GB	CGH	Patients with both chromosome 7 gain and chromosome 10 loss had the shortest overall survival, while those with copy-number neutral profiles survived longer.
Huhn SL et al. [10]	USA, 1999	Observational study	30	Primary GB	CGH	Radiation-resistant tumors showed more frequent CNAs, suggesting greater genomic instability, but the findings were not statistically significant.

**Table 2:** Summary of clinical outcomes of the included studies.

Authors	Study Population					Management	Outcomes
	Sample Size	Mean Age	EGFR Gene Status	Chromosomal Arrangement	KPS		
Arslantas et al. [7]	20	53.2 ± 10.8	NR	(+7/−10) 5/20	80–100 11/20 ≤70 9/20	Primary surgery 20/20 Reoperation 13/20 Chemotherapy NR Fractionated radiotherapy 14/20	+7/−10 5.0 ± 3.1 months vs. −10 5.4 ± 3.3 months vs. Overall, 10.0 ± 5.6 months survival
Lopez-Gines C et al. [9]	25	55.8	EGFR amp 13/25 +7/−10 + EGFR amp 7/25	(+7/−10) 14/25 (+7/−10) 5/25 (+7/−10) 3/25 (+7/−10) 3/25	80–100 21/25 ≤70 4/25	Primary surgery (subtotal resection) 23/25 Biopsy only 2/25 Chemotherapy 20% of patients Radiotherapy 70% of patients	+7/−10 36 ± 3.7 weeks vs. No alteration 48 ± 4.7 weeks survival
Stichel et al. [8]	167	NR	NR	(+7/−10) 115/167	NR	NR	Similar survival curves among GB with +7/−10 and those without
Nair NU et al. [6]	141	NR	NR	(+7/−10) 111/141 (+7/−10) 6/141 (+7/−10) 20/141 (+7/−10) 4/141	NR	NR	+7/−10 ~70 weeks vs. No alteration ~170 weeks survival
Huhn SL et al. [10]	RR 20 RS 10	RR 47 RS 45	NR	(+7/−10) RR 9/20 (+7/−10) RS 1/10	NR	Subtotal resection RR: 19/20 RS: 9/10 Gross total resection RR: 1/20 RS: 1/10 Radiotherapy RR: 18/20 Single Fraction total dose/day 5800–6100 cGy RS: 7/10 Single Fraction total dose 5800–6100 cGy RR: 2/20 Hyperfractionated total dose/day 7040–7240 cGy RS: 3/10 Hyperfractionated total dose/day 7040–7240 cGy Radiation sensitizers Hydroxyurea and/or Alfa-Difluoromethylornithine RR: 12/20 RS: 7/10	RR group: more CNAs, shorter survival (384 vs. 462 days); no link between CGH findings, BrdUrd index, or survival

EGFR = Epidermal growth factor receptor, EGFR amp = Epidermal growth factor receptor amplification, KPS = Karnofsky performance status, RR = radio-resistant, RS = radio-sensitive, CNAs = Copy-number alterations, NR = not reported, CGH = comparative genomic hybridization.

## Data Availability

No new data were created or analyzed in this study. Data sharing is not applicable to this article.

## Supplementary Materials

The following supporting information can be downloaded at: https://www.mdpi.com/article/10.3390/brainsci16010060/s1, Preferred Reporting Items for Systematic reviews and Meta-Analyses extension for Scoping Reviews (PRISMA-ScR) Checklist. Reference [38] is cited in the supplementary materials.

## Abbreviations

The following abbreviations are used in this manuscript:

GBM	Glioblastoma
Ch+7/−10	Co-occurrence of chromosome 7 gain and chromosome 10 loss
FISH	Fluorescence in situ hybridization
OS	Overall survival
CNS	Central nervous system
IDH_wt_	Isocitrate dehydrogenase wildtype
TCGA	The cancer genome atlas
CNAs	Copy number alterations
SAC	Spindle assembly checkpoint
TERTp	Telomerase transcriptase reverse promoter
